# Supplementary material for: Association of Peripheral Inflammatory Biomarkers and Growth Factors Levels with Sex, Therapy and Other Clinical Factors in Schizophrenia and Patient Stratification Based on These Data
Source: Brain Sci. 2023 May 22;13(5):836. doi: 10.3390/brainsci13050836 (PMC10216189; doi:10.3390/brainsci13050836)
Supplement: Supplementary file 1 [file brainsci-13-00836-s001.zip › Supplement Figures.pdf]

Supplement Figures

Article

Association of Peripheral Inflammatory Biomarkers and Growth Factors Levels with Sex, Therapy and Other Clinical Factors in Schizophrenia and Patient Stratification Based on These Data

Evgeny A. Ermakov [1, 2, *], Mark M. Melamud [1], Anastasiia S. Boiko [3], Daria A. Kamaeva [3], Svetlana A. Ivanova [3], Georgy A. Nevinsky [1, 2], and Valentina N. Buneva [1, 2]

[1] Institute of Chemical Biology and Fundamental Medicine, Siberian Branch of the Russian Academy of Sciences, 630090 Novosibirsk, Russia
[2] Novosibirsk State University, Department of Natural Sciences, Novosibirsk 630090, Russia
[3] Mental Health Research Institute, Tomsk National Research Medical Center of the Russian Academy of Sciences, 634014 Tomsk, Russia
* Correspondence: evgeny_ermakov@mail.ru

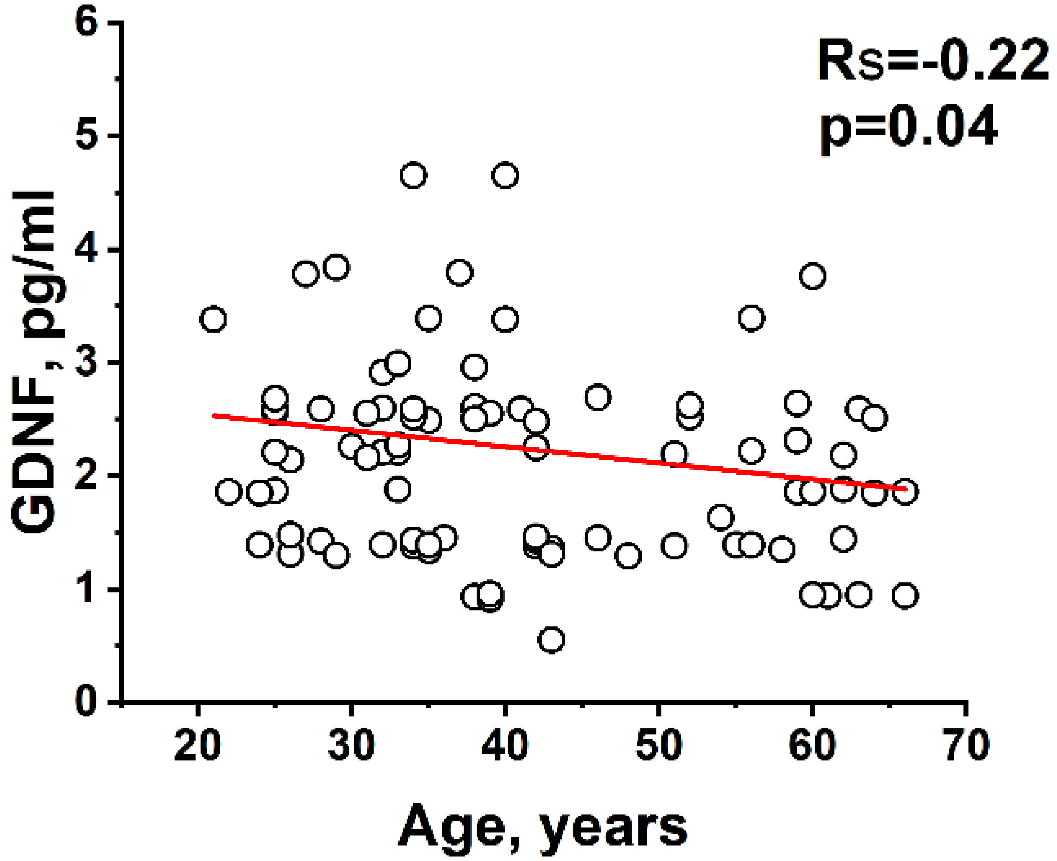

Figure S1. Correlation of serum GDNF level with the age of patients with schizophrenia. Rs is the Spearman correlation coefficient.

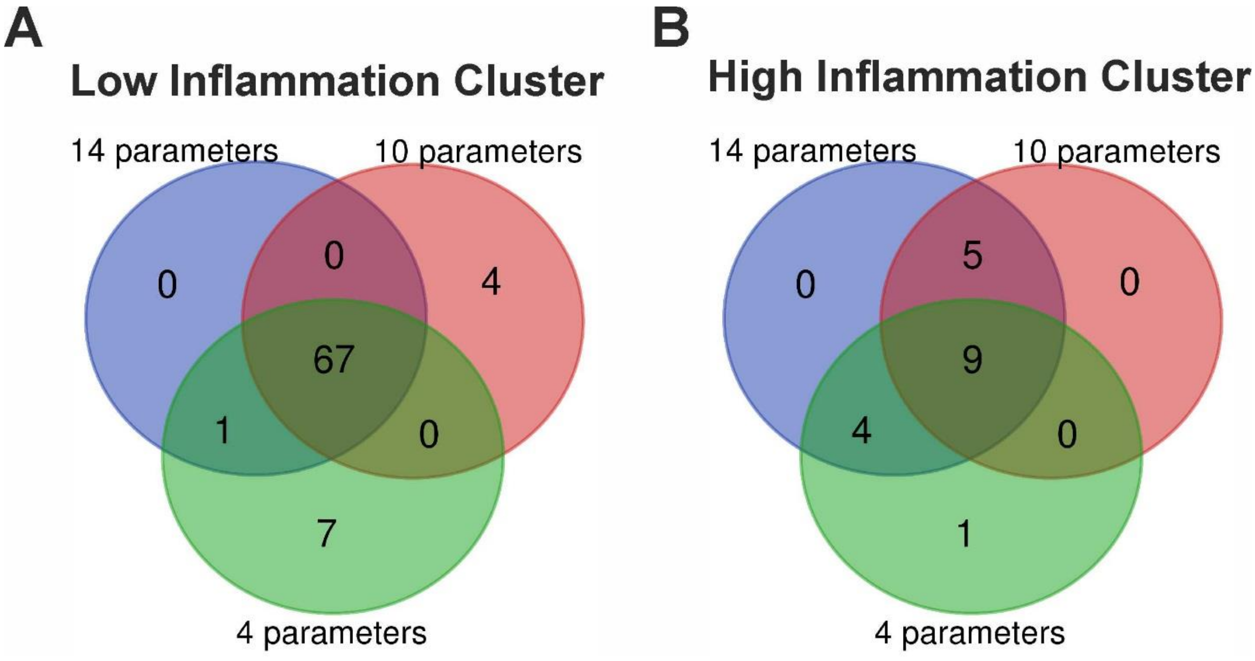

Figure S2. Venn diagram showing the number of patients classified to "Low Inflammation Cluster" (**A**) and "High Inflammation Cluster" (**B**) when clustered using 14, 10 and 4 parameters (cytokine and growth factor levels).
